# Overview on Percutaneous Therapies of Disc Diseases

**DOI:** 10.3390/medicina55080471

**Published:** 2019-08-12

**Authors:** Salvatore Masala, Fabio Salimei, Adriano Lacchè, Stefano Marcia, Francesco Massari

**Affiliations:** 1 S. Giovanni Battista Ordine di Malta Hospital, 00148 Rome, Italy; 2 Department of Diagnostic Imaging and Interventional Radiology, General Hospital, University of Rome, Tor Vergata, 00133 Rome, Italy; 3 ASSL Cagliari, Radiology PO SS Trinità, ATS Sardinia, 08100 Nuoro, Italy; 4 Department of Radiology, University of Massachusetts, 55 Lake Avenue North, Worcester, MA 01655, USA

**Keywords:** intervertebral disc, percutaneous therapies, disc diseases, lumbosciatalgy, low back pain

## Abstract

Low back pain is an extremely common pathology affecting a great share of the population, in particular, young adults. Many structures can be responsible for pain such as intervertebral discs, facet joints, nerve roots, and sacroiliac joints. This review paper focuses on disc pathology and the percutaneous procedures available to date for its treatment. For each option, we will assess the indications, technical aspects, advantages, and complications, as well as outcomes reported in the literature and new emerging trends in the field.

## 1. Introduction

The term low back pain (LBP) describes a clinical entity characterized by algic symptoms in the lumbar region that hamper normal daily activities, with consequences on overall quality of life [1].

It is estimated that about 80% of all people, during the course of their lives, will suffer from at least one episode of LBP, with a one-year prevalence rate in the US between 5–20% [1,2].

In the great majority of cases, it constitutes an isolated and self-limited event as testified by complete functional recovery rates, with a rest-only approach and no treatment, of 60% at 1 week and 95% at 12 weeks. However, there is a high incidence of relapsing episodes and, in some cases, the duration of symptoms can exceed 12 months, which is identified as “chronic low back pain” [2].

Low back pain, mainly chronic disease, carries enormous social and economic costs; it represents the most frequent cause of disability among young adults (age < 45 years) and is thus the first cause of absence from work [1,3].

In the settings of LBP, there are a few structures known to act as pain generators; a great share of events is usually caused by intervertebral disc disease, nerve root inflammation, and/or zygapophyseal joint degeneration. Degenerative disc changes are usually associated with vascular and nervous fiber infiltration from the outer portions of the annulus fibrosus (AF) into the deeper structures of the disc, leading to symptoms; on the other hand, a bulging disc may cause compression and, therefore, inflammation of the adjacent nerve root, resulting in neurogenic pain from the affected fibers [3].

The sacroiliac joint may also be responsible for lumbar pain in up to 15–30% of cases, usually located below the L5 level [4].

For the purposes of this article, we will focus on disc pathology, its clinical presentation, and the current treatments available. 

The intervertebral disc is responsible for up to 40% of LBP cases; that percentage is even greater among young adults (<45), where the disc is the first structure involved following stressful mechanical events acting on the spine [4].

Kirkardy Willis first described disc degeneration as a key component of LBP in 1983, introducing the concept of mechanical instability [5].

Yet, before a mechanical deficit, the disc goes through a complex series of biological changes, mainly due to metabolic dysfunction of the nucleus pulposus (NP) cells, the main producers of the disc extracellular matrix [6].

The matrix is continuously produced and reabsorbed by the cells of the NP, in a constant equilibrium between lytic enzymes (MMP and ADAMS) and growth factors (BMP, GDF). Inflammatory molecules, such as TNF-a and IL-1, also promote degradation of the matrix [7].

Repeated mechanical solicitations lead to increased reabsorptions of the matrix and a poor production in terms of its quality. This causes an unbalanced force distribution, extending the disease to the AF, because of the inability to withstand the mechanical load generated through daily activities alone [8].

The genesis of discogenic pain (low back pain and neuralgia) is related to herniation of the intervertebral disc, caused by annulus fibrosus fissuration with the subsequent release of nucleus pulposus. Histopathophysiological effects include the mechanical pressure effect, inflammatory reaction, and neovascularization [9].

Mechanical effects cause direct pressure on nerve roots or on vessels, causing ischemia and inflammatory reactions, which stimulate the immune system in producing phospholipase A2, prostaglandin, leukotriene, and matrix metalloproteinase [10].

Proposed treatments for such conditions include conservative therapy, injections, and percutaneous and surgical procedures. 

Minimally invasive percutaneous interventions, apart from epidural injections of corticosteroids and analgesics, include decompression techniques (mechanical, thermal, or chemical), biomaterial implantation, and disc cell therapies.

In this paper, we will review the technical aspects and clinical outcomes of the main percutaneous procedures available today. 

## 2. Techniques

In the settings of LBP, the standard approach is a 4–6 week conservative therapy course with analgesics, NSAIDs, physical therapies, and/or bracing with a combination of epidural injections. Percutaneous injections, in association with physiotherapy, can be performed as an intermediate step between conservative therapy, percutaneous decompression techniques, and surgical options. Percutaneous therapies include injections via an interlaminar, caudal, or transforaminal approach; mechanical, thermal or chemical decompression; and biomaterial implantations/disc cell therapies [9,11,12,13,14,15,16,17,18] (Table 1).

### Mechanical, Thermal, or Chemical Decompression Techniques

A patient primary inclusion criterion for percutaneous therapies is the presence of discogenic pain (low back pain and neuralgia) from an intervertebral disc herniation that occupies less than one-third or half of the canal diameter in MRI that has also failed to resolve after conservative therapy for about 4–6 weeks and at least one session of steroid injection. Symptoms have to be consistent with the segmental level where the herniation is present in MRI [9,11,14,15,19].

Absolute contraindications for the percutaneous technique, which prompt emergent surgical management, include sphincter dysfunction, extreme sciatica, and a progressive neurologic deficit, along with the impossibility of obtaining patient-informed consent. Other contraindications are asymptomatic herniation, sequestered disc fragment, local or systemic infection, and spondylolisthesis [9,11,14,15,19]. In the case of hemorrhagic diathesis alterations, those must be corrected, and anticoagulant therapy should be interrupted according to international guidelines.

All the procedures are primarily performed via fluoroscopic guidance, although CT and MRI are both viable [9,11,14,15,20]. Recently, an endoscopic fiber-optic approach was proposed [21].

**Figure 1:**
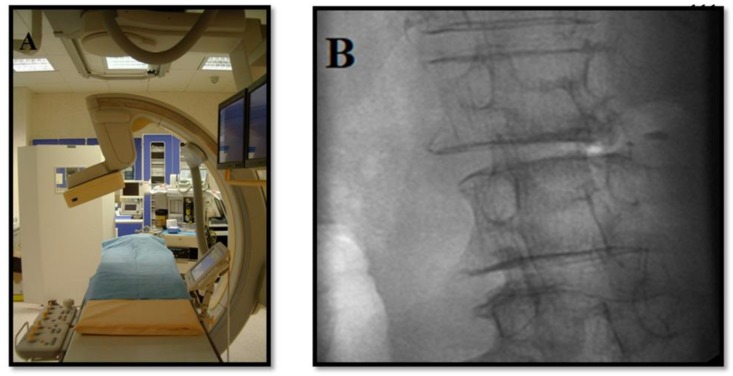
Procedure under fluoroscopic guidance. (**A**) Angiographic suite. (**B**) Oblique (~45°) “Scottie-dog” projection.

**Figure 2:**
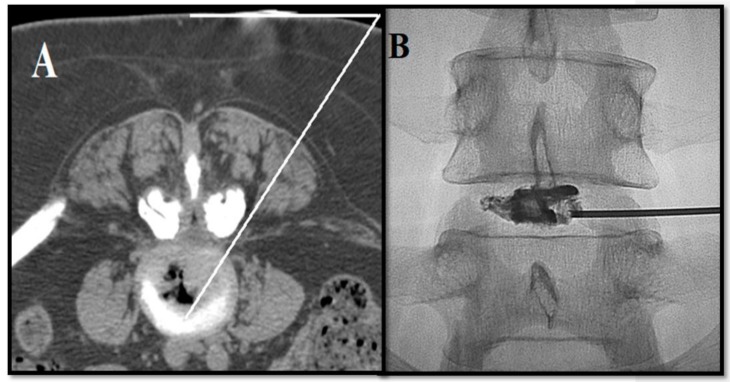
(**A**) Needle trajectory obtained via CT guidance. (**B**) Patient with symptomatic L4–L5 intervertebral disc herniation. Antero-posterior fluoroscopic view: discography shows correct needle positioning.

With the sterile technique, in accordance with the Cardiovascular and Interventional Radiological Society of Europe Standards of Practice for percutaneous treatment of intervertebral disks [22], the patient is placed in the prone position. Whenever fluoroscopy is used as a guiding modality, the target disc should be centered and vertebral end plates aligned in the A–P projection. Rotation of the fluoroscopy beam at ~45° (the spinous process should point toward the contralateral facet joint) produces the “Scottie dog” projection (Figure 1). In this projection, under continuous fluoroscopy, the trocar, usually an 18-G spinal needle with variable length (9–15 cm), is advanced until the periphery of the disc is reached. Fluoroscopic projections in the A–P and lateral projection are used to verify the proper trocar positioning. Then, in the lateral projection, the trocar is advanced within the disc. The final position of the needle tip should be within the anterior third of the disc space in the lateral projection and toward the midline in the A–P projection midway between the two endplates (Figure 2). Whenever computed tomography is used as a guiding modality, the access point on the skin is selected and marked. While checking the correct path with sequential scans, the trocar is advanced within the disc utilizing a posterolateral approach through the aforementioned route. For the L5–S1 intervertebral disc, alternative techniques include the use of curved trocars or posterior extra-thecal access through the lateral epidural space. When the correct positioning is obtained and verified, decompression is performed through the trocar.

Cervical intervertebral discs are usually treated utilizing an antero-lateral approach with the patient in the supine position. The trocar is advanced, with prior subluxation of the larynx, between the larynx and jugular–carotid vessel. Due to the presence of the esophagus on the left side, the right-sided approach is preferred. When fluoroscopic guidance is used, the correct position is deemed to be within the posterior third in the lateral projection and, as for the lumbar discs, toward the midline in A–P projections. With CT guidance, the entry point on the skin is marked and the trocar is advanced through the route described before [17].

**Figure 3:**
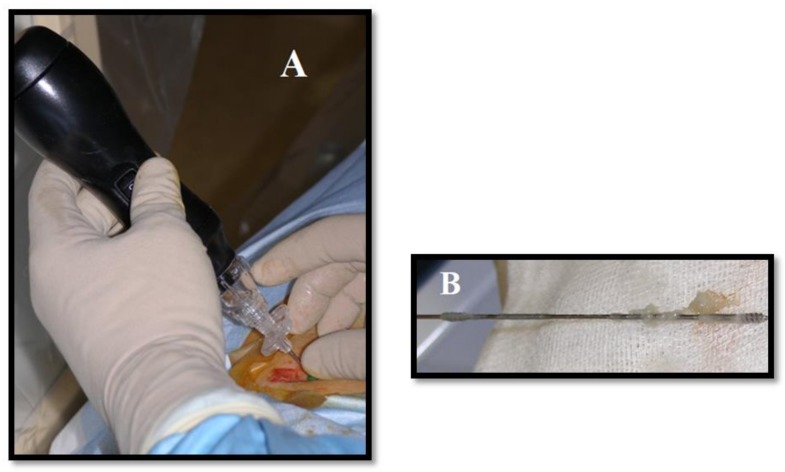
Mechanical decompression of herniated L4–L5 disk. (**A**) Decompression device (mechanical high-rotation-per-minute device with spiral tips) is placed via postero-lateral access under fluoroscopic guidance. (**B**) Part of the disk is removed.

Mechanical percutaneous decompression devices include high-rotation-per-minute (RPM) devices with spiral tips, metallic wires/laminae, or water/pneumatically driven suction-cutting probes, which remove approximately 1–3 g of disc material anterior to herniation via a 17-G cannula in the lumbar disc and 19-G in the cervical disc; some of those devices use the Archimedes’ screw principle to extract material from the nucleus pulposus (Figure 3) [9,11,14,15]. Endoscopic discectomy is performed via a postero-lateral route, which provides a way to decompress nerve roots by aspiring the herniated part of the disk [23].

Thermal percutaneous techniques inhibit intradiscal cytokines that are associated with intervertebral disc degeneration, destroy nociceptors in the periphery of the annulus, and fuse collagen annular fibers with resultant shrinkage at the disc periphery [11].Those thermal techniques include lasers, radiofrequency (with continuous or pulsed RF energy delivery), and nucleoplasty [9,14,15,20,24,25,26].

Even if percutaneous laser disc decompression (PLDD) can be performed with different kinds of lasers (diode, Nd:YAG, KTP, CO_2_, Ho:YAG), the therapeutic principle is the same: By introducing a laser probe (0.4mm) within the middle of the intervertebral disc, a small volume of nucleus pulposus (mainly composed of water) is vaporized by the energy generated by the laser beam. This reduces intradiscal pressure and subsequently produces retraction of the herniated component toward the center of the disc. To avoid risk of complications, pulsed laser energy delivery is necessary, in order to allow for dissipation of the heat generated after every single pulse and before administration of the next one. There are no statistically significant differences in outcomes and complications between those different varieties of lasers, with the exception of CO_2_, which requires a metal cannula for laser beam administration, whose heating may cause thermal nerve root damage [27].

Continuous RF (CRF) uses high-frequency EM waves to induce coagulative necrosis in target tissue, which occurs at temperatures between 60 °C and 80 °C. Pulsed RF (PRF) uses radiofrequency current in short, high-voltage bursts followed by a “stop” phase that allows heat elimination, generally keeping tissue at a temperature of ~40 °C. While the former generates necrosis in the target tissue, PRF seems to involve a temperature-independent pathway by rapidly changing the electrical field, which influences the production of anti-inflammatory cytokines [24,28].

**Figure 4:**
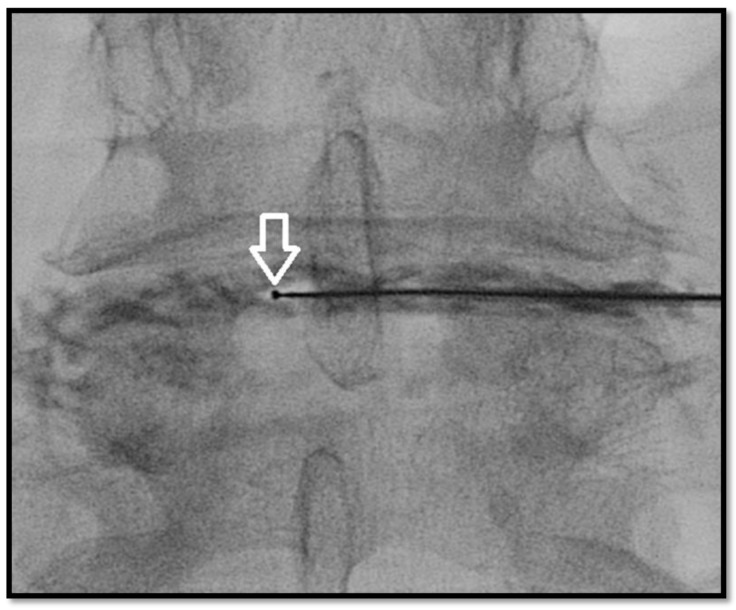
Antero-posterior fluoroscopic view. L4–L5 intervertebral disc nucleoplasty: tip of the wand (white arrow) during ablation, performed clockwise.

The nucleoplasty wand, via low-temperature plasma field-controlled ablation, channels the postero-lateral to the antero-medial aspect of the annulus, with minimal risk of thermal injury. During advancement and sequential clockwise rotation of the wand, the ablation creates six channels at the 2, 4, 6, 8, 10, and 12 o’clock positions, at a speed of 0.5 cm/s using120 volts of energy with resultant temperatures of 50–70 °C. During retraction instead, the coagulation effect denaturizes type II collagen and proteoglycans, shrinking the surrounding collagen and widening the channel (~1mm) (Figure 4) [26].

A comparison between conservative therapy and mechanical decompression favors the latter in terms of efficacy, resulting in more significant and long-lasting pain relief in those patients treated with percutaneous techniques [29].

Chemical percutaneous decompression techniques are mainly based on the utilization of two compounds: the oxygen–ozone mixture and radiopaque gelified ethanol.

**Figure 5:**
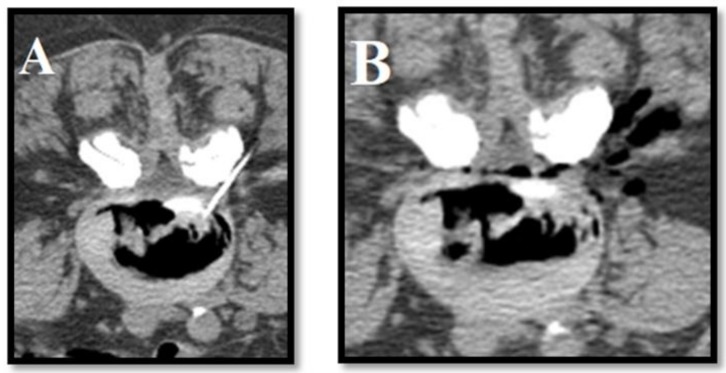
O_2_/O_3_: (**A**) intradiscal and (**B**) peri-radicular injection in patient with right lumbosciatalgia from L3-L4 herniated disk. Postero-lateral access is obtained via CT.

O_2_–O_3_ therapy is based on the injection of a mixture of O_2_ and O_3_ gas inside the nucleus pulposus, in order to reduce the intradiscal pressure, compression on the surrounding structures, and, consequently, associated pain. While withdrawing the needle from its intradiscal position, the gas mixture is often also administered in the soft tissues surrounding the inflamed nerve root (Figure 5) [30,31].

The properties of intradiscal ozone administration are well known in clinical practice [32], ranging from nucleus pulposus depressurization to immuno-modulant effects. Although the biochemical mechanisms at the base are not fully comprehended, recent studies have shown a glycosaminoglycan lysis ozone mediated, which reacts with fragmented proteoglycans in causing dehydration of the disc; an interaction was also observed with the intradiscal cytokines, generating an anti-inflammatory response [31,33].

O_2_–O_3_therapy currently constitutes one of the most used techniques in symptomatic disc herniation.

Radiopaque gelified ethanol intradiscal administration represents an emerging minimally invasive procedure in the management of disc pathology; the effect of ethanol on biological tissues has long been known and used by many interventionists during their procedures. The adjunction of ethyl-cellulose and tungsten provides more viscosity (to reduce diffusivity) and radiopacity, respectively, to the compound [34,35].

**Figure 6:**
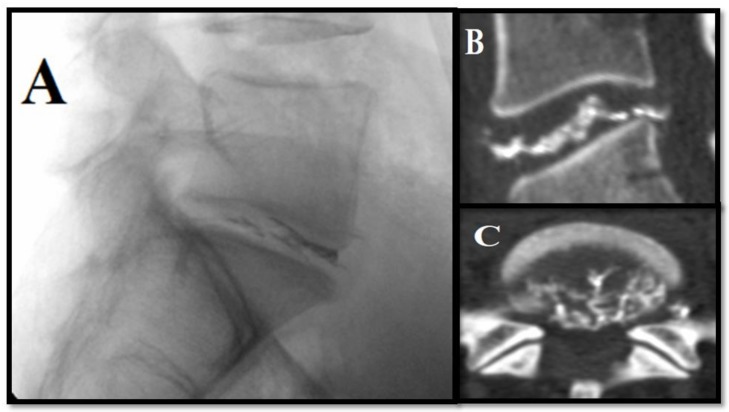
Radiopaque gelified ethanol for L5–S1 disc herniation: (**A**) Lateral fluoroscopic view: notice radiopaque gel in the L5–S1 disc. (**B**,**C**) Post-intervention follow-up CT exam shows optimal intradiscal distribution of the solution.

**Figure 7:**
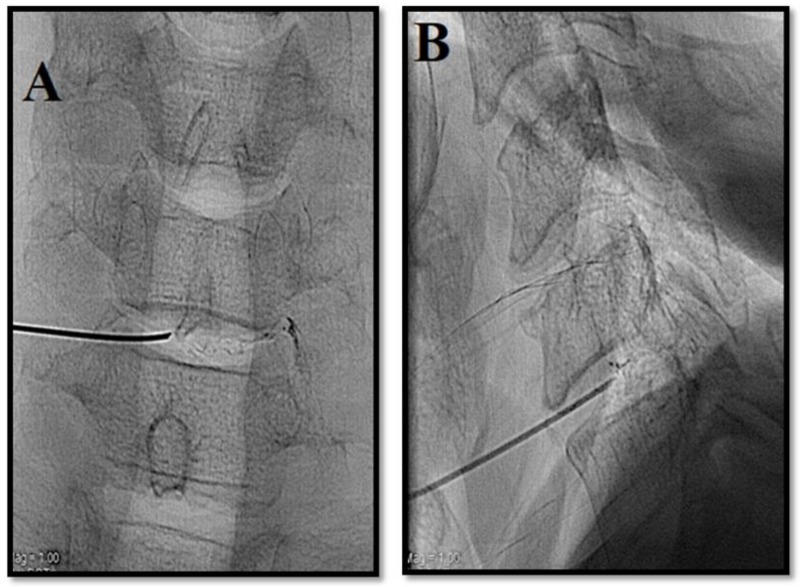
Radiopaque gelified ethanol for cervical disc pathology. Patient with symptomatic C5–C6 intervertebral disc herniation. (**A**,**B**) Antero-posterior and lateral fluoroscopic view. Access to the disc is obtained with an antero-lateral approach under fluoroscopic guidance prior to subluxation of the larynx, between the larynx and jugular–carotid vessel. In the cervical tract, a lower quantity of gel is usually injected (ca. 0,3 mL).

The solution produces local necrosis within the nucleus pulposus resulting in dehydration and mechanical retraction of the bulging disc [36]. Additionally, the gelified compound has hydrophilic properties, able to generate fluid inflow from the periphery toward the core of the nucleus pulposus; In addition, the deposition and precipitation of the gel particles result in a “prosthesis” effect at the injection site [34]. A total amount of 0.8mL is usually sufficient for the treatment of a single lumbar herniated disc. Its use is also considered effective in the cervical spinal segment (Figure 6 and Figure 7) [37,38].

Percutaneous disk decompression techniques show a pain reduction success rate between 75 and 80%, with long-term stable effects and a significant reduction in the Oswestry disability index (ODI) and visual analogue scale (VAS) values post-procedurally and during follow-up examinations [9,11,14,15,30,32,39,40]: comparative studies among those techniques do not favor one in particular [41,42].

Several randomized prospective trials comparing conservative therapy, infiltrations, and decompression techniques have demonstrated the superiority of the latter in producing a better and longer lasting pain reduction [25,29]. Further trials have been conducted analyzing and comparing the percutaneous decompression, open discectomy, or micro-discectomy procedures, reporting no statistically significant differences in outcomes among these therapies [43,44,45]. Percutaneous techniques, however, compared to open disc surgery, cause only minimal degeneration of the surrounding tissues, in particular, of muscles, which are a pivotal structure in the support of the degenerated intervertebral disc and are considerably more cost-effective (25–30%) [46].

Complications are encountered rarely, with spondylodiscitis being the most severe (0.24%). Iatrogenic complications, such as the puncture of the dural sac and cord and nerve root damage, as well as hemorrhage, are significantly less frequent [1].

Nevertheless, randomized and observational trials are needed to further confirm this evidence [47,48,49].

## 3. Biomaterial Implantationand Disc Cell Therapies

A relatively new approach for degenerative disc disease (DDD) treatment is based on the implantation of biomaterials, in particular, hydrogel-based compounds with the aim of nucleus pulposus regeneration in a disc with a fairly intact annulus fibrosus [50].

In this application, there are no definitive criteria that must be satisfied to achieve procedural success; the current focus is on developing materials with properties similar to those of the native structure, allowing for better integration of implants without displacement, migration, or rupture. In addition, the goal of restoring disc function, as in disc height and range of motion, must be pursued [51,52].

Mainly described in animal models, the clinical translation of implanted biomaterials can only occur with reliable evidence of durability and the ability to maintain their properties and integrity through repetitive solicitation, and the lack of hypothetically provoking a local or systemic immune response [51,53,54].

Proposed biomaterials for DDD can be biologically derived or synthetically produced. The formers (e.g., agarose, alginate, hyaluronate) possess good biocompatibility with the native environment but inferior performances in comparison to synthetic ones, mainly constituting poli-vinyl-alcohol (PVA) and related polymers. As such, a combination of those elements has been tested in many studies in order to improve the mechanical and biochemical properties of the implanted materials [55].

Moreover, biomaterials derived from silk have recently gained attention in this field and are objects of several studies assessing the effectiveness and security of their implantation [56].

Finally, some biomaterials (typically of natural origin, such as hyaluronate and collagen) have been proposed to act only as carriers for cell delivery to the nucleus pulposus, aiming to provide an environment supporting cell-mediated disc regeneration [51,56].

There is a continuously increasing interest in the use of cellular therapies for disc regeneration in the degenerative disc disease. Those mainly consist of the use of platelet-rich plasma (PRP) and stem cells (MSC).

Platelet-rich plasma (PRP) is obtained by concentrating platelets and other blood components from an autologous sample of blood. The platelets, growth factors, and cytokines in the solution augment collagen content and production, accelerate tissue regeneration, and promote angiogenesis [57].

PRP appears to inhibit the inflammatory effect of TNF-alfa and Interleukin-1 on nucleus pulposus cells [58].

Even if multiple studies demonstrate its effectiveness and safety with a modest reduction in VAS and ODI score, no significant results have been observed in the early stages, because of the time required for the treatment effect to occur, although more trials are needed to confirm its effectiveness [59,60].

Stem cell therapy consists of the transplantation of MSCs in the nucleus pulposus of the affected disc. Those cells, capable of aggrecan production, have led to an increase in disc water content and height in human models (demonstrated with MRI) with a sensible improvement in pain and functional status [61,62].

A single study described a clinical improvement at a 1year follow-up after intradiscal injection of MSCs [60].

A major drawback of this procedure is cell extravasation, potentially causing complications such as discitis, tumorigenesis, osteophytes, and spinal stenosis [63,64].

## 4. Conclusions

Percutaneous intervertebral disc procedures are practical and reproducible treatments for symptomatic intervertebral disc herniations that have failed a combination of 4–6 weeks of conservative therapy associated with a session of steroid infiltration, carrying a success rate of 75–80% and rare complications (~0.5%), with spondylodiscitis being the most severe (0.24%). Sterile techniques and prophylactic antibiotics are a prerequisite. Imaging guidance allows clinical success with a markedly decreased complication rate.

There is no definitive evidence regarding the difference in efficacy between surgical options and the percutaneous decompression technique, although the latter results in only minimal destruction of the surrounding structures, in particular, of muscles, which support the altered intervertebral disc, and are considerably more cost-effective.

In conclusion, percutaneous intervertebral disc therapeutic techniques are viable as a valid treatment before surgery, for the treatment of symptomatic herniation of both the cervical and lumbar spine.

## Figures and Tables

**Table 1:** Percutaneous intervertebral disc techniques

Technique	Method
Mechanical decompression	Mechanical high RPM spiral tips or metallic laminae probes
	Water-driven suction-cutting probe
	Pneumatically driven suction-cutting probe
	Herniotome
Thermal decompression	Percutaneous laser decompression
	Intradiscal electrothermaltherapy
	Intervertebral disc nucleoplasty
	Pulsed radiofrequency
Chemical decompression	Radiopaque gelified ethanol
	Ozonetherapy
Biomateral implantation	Hydrogel
Cellular therapies	Platelet-rich plasma, stem cell therapy
